# Supplementary material for: TMEM106B C-Terminal fragment aggregation exacerbates α−synuclein and TDP-43 proteinopathy phenotypes
Source: bioRxiv. 2026 Oct 30:2026.10.29.762961. Preprint. [Version 1] doi: 10.64898/2026.10.29.762961 (PMC13734141; doi:10.64898/2026.10.29.762961)
Supplement: Supplementary file 1 [file NIHPP2026.10.29.762961v1-supplement-1.pdf]

bioRxiv preprint doi: https://doi.org/10.64898/2026.10.29.762961; this version posted October 30, 2026. The copyright holder for this preprint (which was not certified by peer review) is the author/funder. This article is a US Government work. It is not subject to copyright under 17 USC 105 and is also made available for use under a CC0 license.

Supplemental Figures

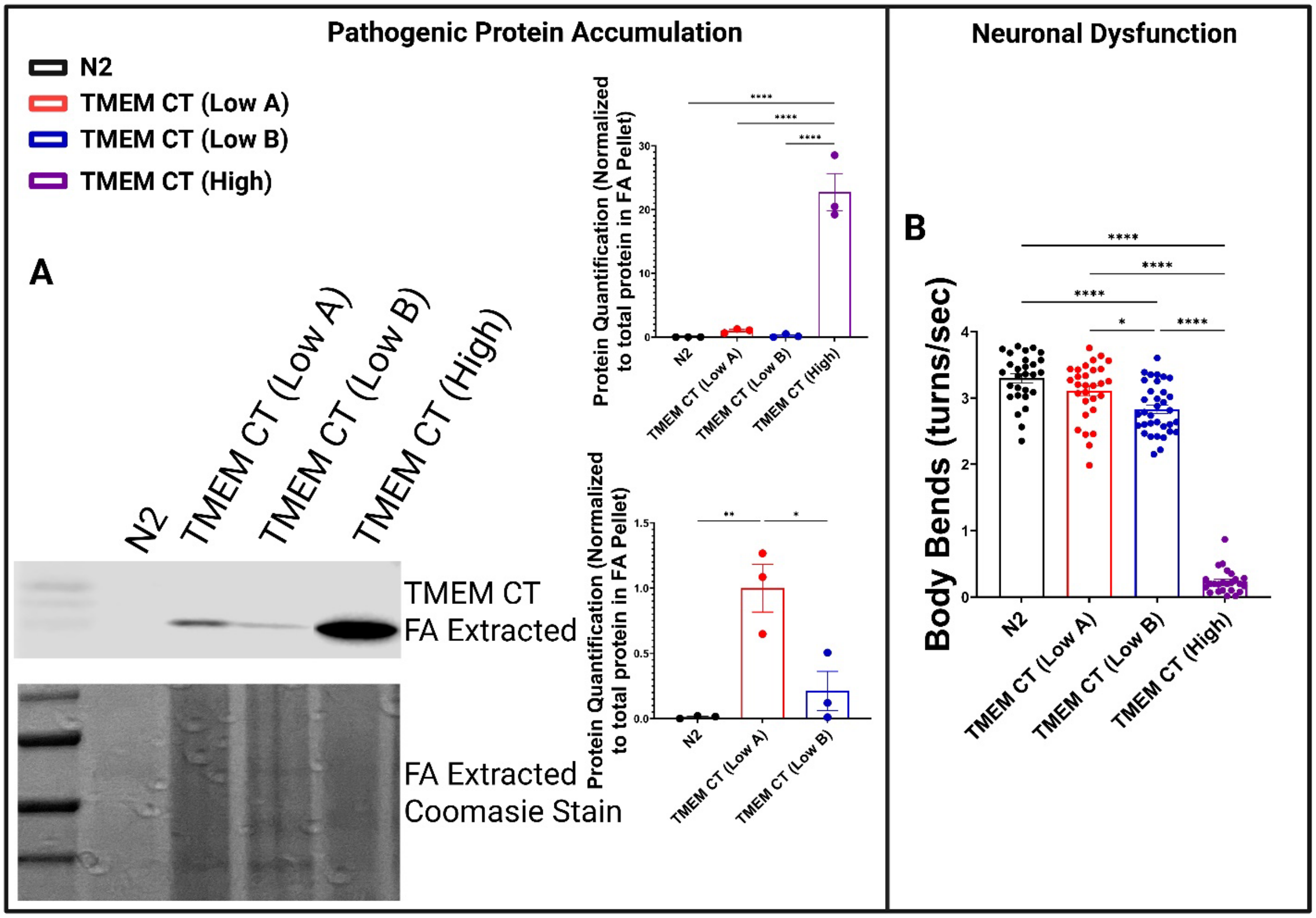

Supplemental Figure 1. Protein accumulation and neuronal function of low expressing TMEM CT *C. elegans* strains. Comparison of non-transgenic *C elegans* (N2), low expressing transgenic *C elegans* (TMEM CT (Low A) or TMEM CT (Low B)), and high expressing transgenic *C. elegans* (TMEM CT (High)). **A)** Western blot analysis and quantification of *C. elegans* populations against TMEM CT (SYSY TMEM106B – 506 017) for detergent insoluble / FA treated samples. N = 4 for each strain, * = P < 0.05, ** = P < 0.001, **** = P < 0.00001. **B)** Liquid thrashing assay of motor neuron function at day 1 of adulthood, measuring body bends for each worm over a 60 s period. n > 30 for each strain, * = P < 0.05, **** = P < 0.00001.

bioRxiv preprint doi: https://doi.org/10.64898/2026.10.29.762961; this version posted October 30, 2026. The copyright holder for this preprint (which was not certified by peer review) is the author/funder. This article is a US Government work. It is not subject to copyright under 17 USC 105 and is also made available for use under a CC0 license.

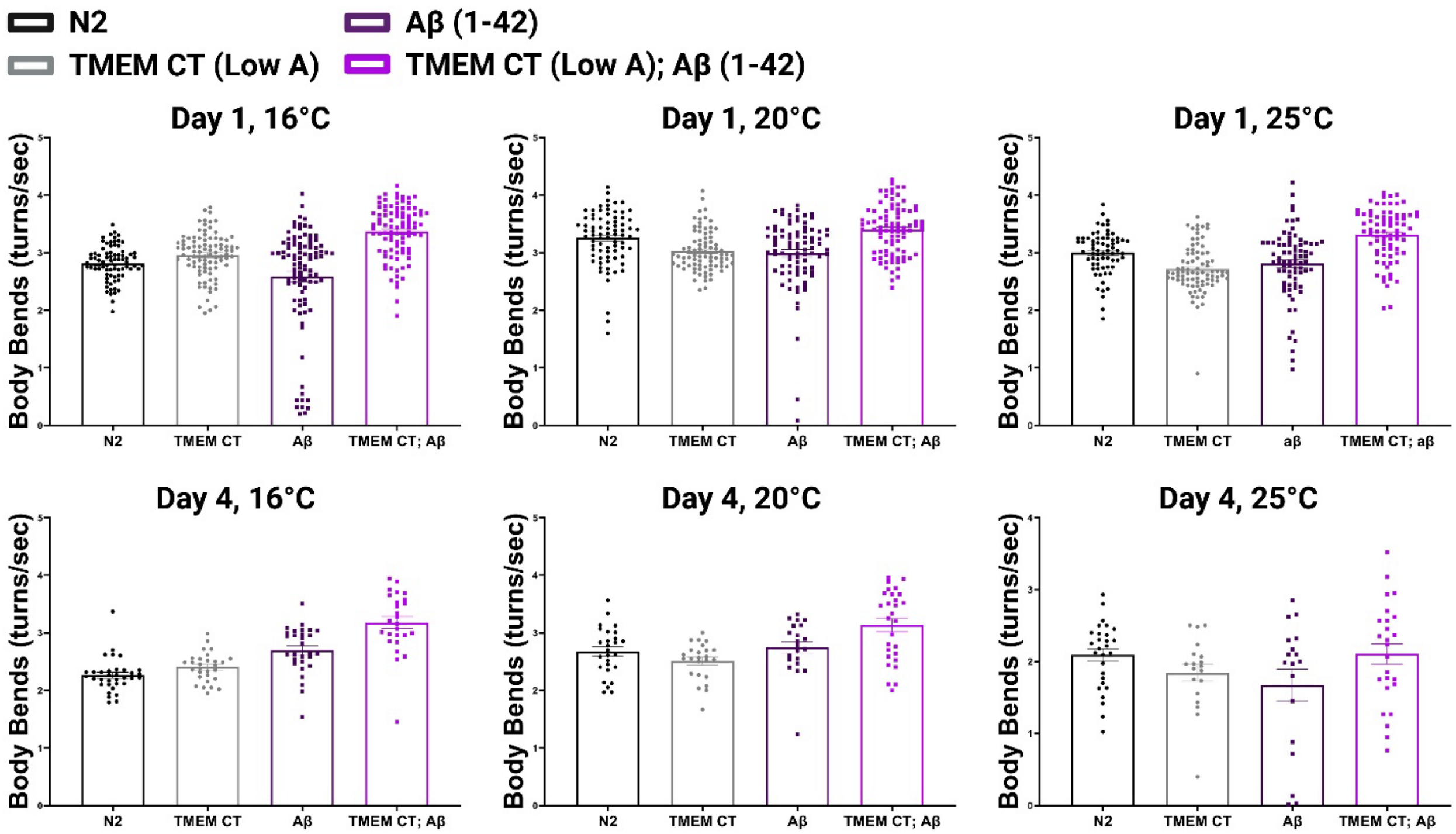

Supplemental Figure 2. TMEM CT aggregation does not promote neuronal dysfunction synergistically with $A\beta_{1\text{-}42}$. Comparison of non-transgenic *C elegans* (N2), single transgenic *C elegans* (TMEM CT (Low A) or $A\beta_{1\text{-}42}$), and bigenic *C. elegans* (TMEM CT (Low A); $A\beta_{1\text{-}42}$). Liquid thrashing assay of motor neuron function at day 1 of adulthood, measuring body bends for each worm over a 60 s period. n > 60, N = 3 for each strain. Liquid thrashing assay of motor neuron function at day 4 of adulthood, measuring body bends for each worm over a 60 s period. n > 20 for each strain.

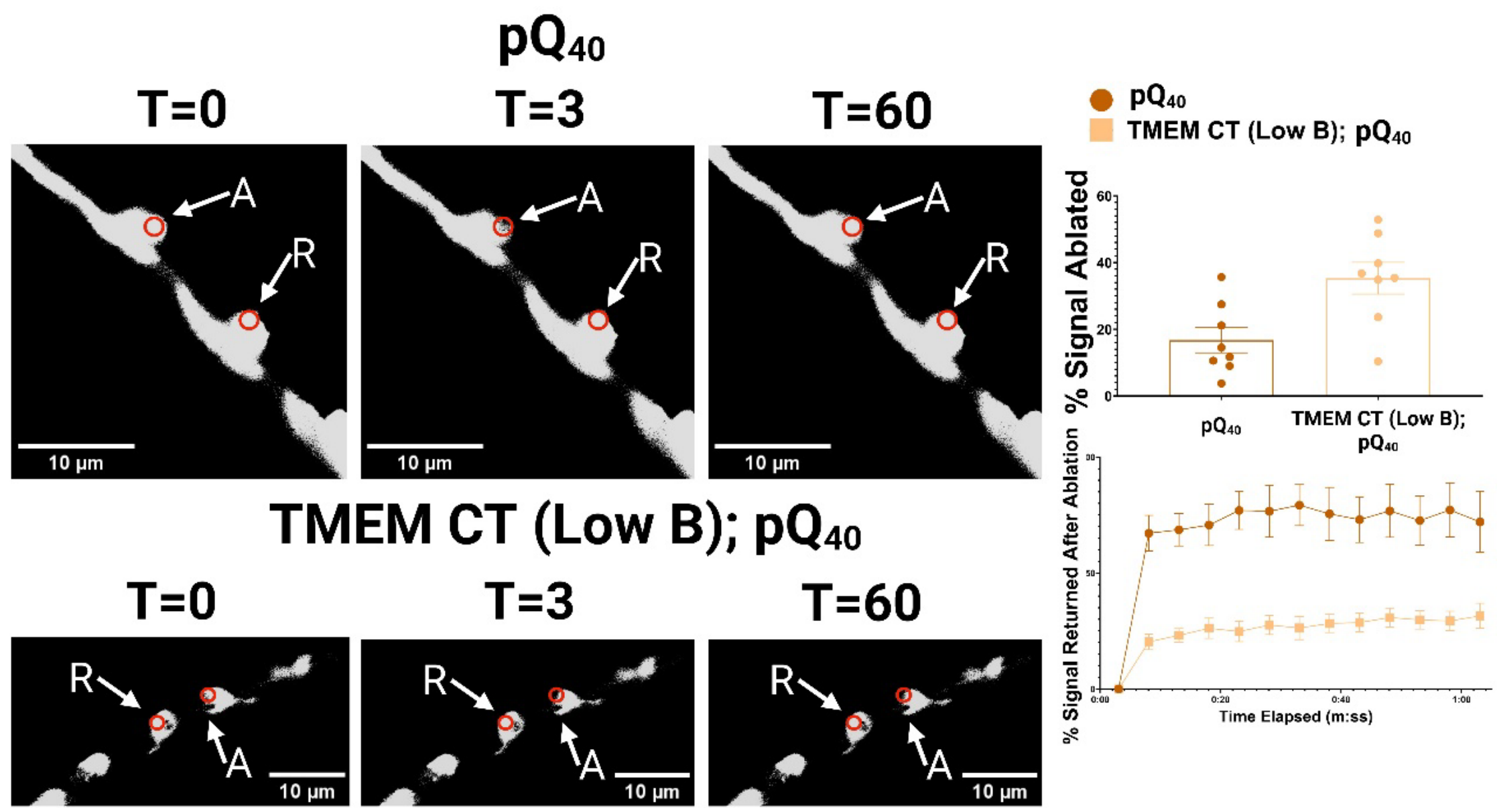

Supplemental Figure 3. TMEM CT aggregation decreases mobility of $polyQ_{40}$. (Left) 100 X magnification of ablated and reference cells or puncta. The left column represents images before ablation, the middle column represents images immediately after ablation, and the right column represents images 1 minute post ablation. Size bar represents 10 µm. Images taken by Nikon Confocal Microscope and processed in FIJI:imageJ. **(Top Right)** Observation of % signal reduction by photobleaching comparing YFP signal of pQ_{40} to TMEM CT (Low B); pQ_{40} (n = 6-8 cells, error bars

bioRxiv preprint doi: https://doi.org/10.64898/2026.10.29.762961; this version posted October 30, 2026. The copyright holder for this preprint (which was not certified by peer review) is the author/funder. This article is a US Government work. It is not subject to copyright under 17 USC 105 and is also made available for use under a CC0 license.

represent SEM). **(Bottom Right)** FRAP analysis comparing recovery of YFP fluorescence after photobleaching. Red circles indicate ablated or reference areas.
